# Associations between maternal adverse childhood experiences and children's physical health conditions: A longitudinal study

**DOI:** 10.1016/j.puhip.2026.100779

**Published:** 2026-03-29

**Authors:** Stefan Kurbatfinski, Janelle Boram Lee, Nicole Letourneau

**Affiliations:** a Department of Community Health Sciences, Cumming School of Medicine, University of Calgary, Calgary, AB, T2N 1N4, Canada; b Alberta Children's Hospital Research Institute, Owerko Centre, Calgary, AB, T2N 1N4, Canada; c Faculty of Nursing, University of Calgary, Calgary, AB, T2N 1N4, Canada; d Departments of Pediatrics, Psychiatry, and Community Health Sciences, Cumming School of Medicine, University of Calgary, Calgary, AB, T2N 1N4, Canada; e Hotchkiss Brain Institute, University of Calgary, Calgary, AB, T2N 1N4, Canada

**Keywords:** Mothers' adverse childhood experiences, Physical health conditions, Logistic regression, Negative binomial regression, The APrON study, Anemia

## Abstract

**Objectives:**

Maternal adverse childhood experiences (ACEs), defined as negative events experienced before 18 years of age (e.g., physical abuse), have been linked to intergenerational impacts on children's physical health. However, research examining associations between maternal ACEs and preschool children's non-psychiatric neurological, respiratory, sensory, digestive, immune, renal, and hematologic conditions remains limited or non-existent, particularly in Canada. This novel Canadian study examined these associations in preschool-aged children.

**Study design:**

This study used data from the longitudinal APrON Study.

**Methods:**

Mothers' ACEs were measured at 12 months postpartum using the ACEs Questionnaire. Mothers reported on proxies of children's non-psychiatric neurological, respiratory, sensory, digestive, immune, renal, and hematologic (indexed by anemia) conditions experienced between 0 and 60 months postpartum using a 16-item, yes/no questionnaire. Unadjusted and adjusted regression models examined associations between maternal ACEs and eight child physical health outcomes (seven domains and a total condition count).

**Results:**

Maternal ACEs were associated with children's hematologic conditions in unadjusted (OR = 1·37, 95% CI [1·06, 1·72]) and adjusted (OR = 1·45, 95% CI [1·10, 1·86]) analyses. However, the associations were attenuated after correction for multiple testing. In both adjusted and unadjusted analyses, maternal ACEs were not associated with non-psychiatric neurological, respiratory, sensory, digestive, immune, renal and total physical health conditions.

**Conclusions:**

Findings contribute preliminary evidence across physical health outcomes and suggest a potential association with children's hematologic conditions, warranting further investigation. Additionally, they suggest that maternal ACEs may not confer substantial intergenerational risk for a range of physical health outcomes in relatively low-risk populations.

## 1 Introduction

Canada, the setting of this study, ranked 30th out of 38 countries for children's physical health in a 2020 World Health Organization report, revealing potential repercussions among Canadian children [1]. The presence of one or more physical health conditions in children, such as non-psychiatric neurological, respiratory, sensory, digestive, immune, renal, and hematologic conditions, are all associated with acute and chronic comorbidities including, but not limited to, mental health conditions, poor educational attainment, and increased healthcare and social services utilization [2,3]. Physical health in childhood is therefore an important indicator of lifelong quality of life and premature mortality [4]. Although genetics, diet, and physical activity are known to influence children's physical health [[5], [6], [7], [8]], [[5], [6], [7], [8]] recent research has focused on the intergenerational effects of parental adverse childhood experiences (ACEs) [9]. Preschool children are especially vulnerable to the negative, intergenerational effects of parental ACEs during this period as it is characterized by rapid physical development [10].

Parents who are exposed to ACEs, defined as adverse events experienced before the age of 18 years including physical, emotional, and sexual abuse and or neglect, parental incarceration, loss, divorce, or separation, and parental substance use or mental health conditions, are at risk of experiencing suboptimal physical health themselves [11]. In one meta-analysis of 37 studies, four or more ACEs were associated with obesity, diabetes, poor self-rated health, cancer, heart disease, respiratory disease, liver or digestive disease, and sexually-transmitted infections [11]. Most of these associations were also described as strong, increasing the potential for causality to be inferred [11].

The numerous health sequelae that parents may experience vis-à-vis ACE exposure can undermine parent-child interaction quality, result in the direct transmission of ACEs to their own children, or perpetuate conditions (e.g., high familial stress) which exacerbate their preschool children's risk of experiencing physical health conditions [[5], [12]]. Mothers exposed to ACEs are more likely to experience chronic stress or socioeconomic disadvantages (e.g., low quality healthcare) during pregnancy which can expose the fetus to suboptimal prenatal conditions [5]. Taken together, all these factors can negatively affect preschool children's development and lead to downstream physiological changes that affect physical health. These pathways likely operate in an integrated and interrelated manner, whereby maternal ACEs influence children's physical health through a combination of biological (e.g., stress physiology), behavioral (e.g., parent-child interactions), and sociodemographic-related mechanisms that together shape early developmental environments.

Using a Chinese sample, Yang et al. (2023) [13] demonstrated a positive association between maternal ACEs and preschool children's overall physical health; however, overall physical health was measured as a single item of children's physical health by mothers using a Likert scale of 1 to 5. In a Canadian sample, a direct effect of maternal ACEs on children's overall physical health (measured as a single item) was not supported [14]. In terms of associations with specific conditions known to reduce children's quality of life, including non-psychiatric neurological, respiratory, sensory, digestive, immune, renal, and hematologic conditions, research remains limited. For example, associations between maternal ACEs and children's non-psychiatric neurological, renal, and hematologic (indexed by anemia) conditions remain unstudied while those with respiratory, sensory, digestive, and immune conditions are only starting to emerge, showing positive associations with mothers' ACEs [[15], [16], [17], [18], [19], [20]]. A description of each condition along with evidence of their associations is provided (Table 1).

**Table 1:** Description and available evidence for each physical health conditions of interest.

Physical Health Condition	Description and Impacts on Children	Evidence of Associations with Mothers' ACEs
*Non-Psychiatric Neurological*	• Non-psychiatric neurological conditions are defined as conditions impacting the nervous system but not leading to mental health symptoms, with high incidence among children [21]. • Non-psychiatric neurological conditions can affect children's executive functioning, muscle movement, regulation of bodily functions, and cognition, all of which are extremely important for children to successfully navigate recreational, educational, and relational contexts [21].	None
*Respiratory*	• Respiratory conditions include illnesses that lead to airway obstructions, targeting the lungs and airway pathways [22]. • Respiratory conditions can be acute, chronic, mild, or severe, leading to lower breathing capacity and oxygen intake [22]. • Certain respiratory conditions, when left untreated, can lead to lower energy levels, increased vulnerability to infections, and at-worst, death, in children [22].	• Condon et al. (2019) [18] found a weak association (trending to significance) between mothers' emotional abuse history and child asthma history (full range of traditional ACEs was not used). • Jones et al. (2019) [16] did not find any significant correlations between mothers' ACEs and different proxies of their children's respiratory sinus arrhythmia. • Tomfohr-Madsen et al. (2016) [15] revealed a greater likelihood of children being diagnosed with asthma if their mothers reported ACEs compared to those without ACEs. • Maternal ACEs resulted in higher odds of asthma diagnosis among children [20].
*Sensory*	• Sensory processing conditions related to hearing, seeing, tasting, smelling, and touching impair children's capacity to intake information from the external environment and properly organize it [23]. • Hearing and seeing are particularly important sensory processing conditions as they are related to communication, undermining their capacity to successfully engage in daily routines and in various settings unequipped to support these communication barriers [23].	• Demers et al. (2024) [19] observed significant associations between mothers' ACEs and their children's visual processing.
*Digestive*	• Digestive conditions in children can entail difficulties in metabolism, nausea, vomiting, irregular bowel movements, and irregular weight changes [24]. • Since digestive conditions can affect nutrient intake, an important factor related to growth and development, they are associated with a host of later physical and health conditions which interfere with children's functioning and lower quality of life [24].	• Maternal physical abuse in childhood was associated with higher risk of obesity in their children [17]. • Maternal ACEs resulted in higher odds of obesity among children [20].
*Immune*	• Immune-related conditions are often governed by a surplus or lack of inflammatory markers which help to fight various viruses, bacteria, and other toxicants [5]. • Children who have suboptimal immune-related functioning are at-risk of experiencing fatigue, chronic illness, allergies, inflammation, autoimmune disorders, and increased healthcare utilization, all undermining lifelong quality of life (e.g., the ability to perform effectively in school) [5]. • Immune-related conditions can also lead to a host of impacts on other physiological systems, such as the digestive and respiratory systems [5].	• Condon et al. (2019) [18] found a weak association (trending to significance) between mothers' history of physical abuse and children's history of allergies (full range of traditional ACEs was not used). • Tomfohr-Madsen et al. (2016) [15] revealed a greater likelihood of children being diagnosed with allergies if their mothers reported ACEs compared to those without ACEs.
*Renal*	• Renal conditions involve irregular kidney functioning or failure, generally stemming from more common, treatable infections to those that are chronic [25]. • Untreated renal conditions can lead to swelling, irregular urination patterns, high blood pressure, growth, and other more common side effects (e.g., fever, fatigue) [25]. • Renal conditions are usually slow progressing, leading to undertreatment at early stages [25].	None
*Hematologic (Anemia)*	• Anemia is the most common hematologic condition in children, that is, a condition which is related to blood or blood-producing organs [7]. • Anemia can be acquired or congenital and either acute or chronic, leading to symptoms of fatigue, cognitive impairment, and poorer performance in school settings [7]. • Prenatal and postnatal nutritional deficiencies, comorbidities, and environmental factors (e.g., toxins) can all exacerbate a child's risk of developing anemia [7].	None

Children's physical health is further shaped by a range of biological, psychosocial, and sociodemographic factors operating during pregnancy and in early childhood. Labor outcomes including gestational age and birthweight, in addition to early caregiving contexts including parenting behaviours, maternal mental health, and maternal social support, have been associated with variation in children's health outcomes [1,26]. Sociodemographic characteristics, including maternal self-identified ethnicity, age, income, and educational attainment, alongside child sex-assigned-at-birth, can further influence children's physical health by shaping their environmental exposures and impacting access to resources and healthcare [1,26]. Therefore, controlling for these theoretically relevant variables when examining predictors of children's physical health outcomes may be important to account for extraneous effects.

Evidently, associations between mothers' ACEs and their preschool children's physical health conditions remain understudied, especially in Canada (with only three Canadian studies identified based on examined literature [14,15,19]). This longitudinal study sought to fill these research gaps by examining associations between mothers' ACEs and their preschool children's physical health conditions, including non-psychiatric neurological, respiratory, sensory, digestive, immune system, renal, hematologic, and total physical health conditions (based on all proxies of each physical health condition) through unadjusted and adjusted models. It was hypothesized that higher maternal ACE scores would be associated with less optimal physical health outcomes in preschool children, specifically, higher odds of non-psychiatric neurological, respiratory, sensory, digestive, immune system, renal, hematologic, and total physical health conditions per maternal ACE exposure. Also, all the outcomes are expected to be dose-dependent, such that increasing numbers of maternal ACEs would be more strongly linked to physical health conditions in children.

## 2 Methods

### 2.1 Study design and setting

This longitudinal study used data from the APrON Study collected across the three trimesters, at three, six, 12, 24, 36, and 60 months postpartum [27]. Information regarding the APrON Study is published elsewhere [27]. The data associated with this study are available upon request from the corresponding author. Ethics was obtained from the University of Calgary Health Research Ethics Board (REB14-1702) and the University of Alberta Health Research Ethics 11 Biomedical Panel (Pro00002954). Informed consent was obtained prior to data collection from all participants with ongoing consent obtained at follow-up data collection points.

### 2.2 Participants

In this study, mothers, who often assume the primary parenting role due to gender expectations and norms and who have been more strongly implicated in children's health outcomes, serve as the focal parent group [28]. Mothers were permitted to participate in this study if: (1) they lived close to Calgary or Edmonton and had the capacity to attend appointments at the Universities of Calgary or Alberta, respectively, until at least three months postpartum; (2) they could speak and read English; (3) were less than 27 weeks gestation prior to entering the study; and (4) were biological mothers of the children [27]. Mothers with twins were excluded from this analysis. A final analytical sample size of 540 was available.

### 2.3 Measurements

**Predictor variable (mothers' ACEs):** Mothers’ ACEs were assessed cross-sectionally at 12 months postpartum using the 10-item, “Yes” or “No”, Adverse Childhood Experiences Questionnaire (Appendix, Figure A) [29]. Despite reliance on recall and limited psychometric validation, the questionnaire is widely used to examine early adversity, consistently demonstrating dose-response associations with various disease risks [[29], [30], [31]].

**Outcomes (children's physical health conditions):** Children's physical health conditions in the following domains were studied: non-psychiatric neurological, respiratory, sensory, digestive, immune, renal, and hematologic based on reports by their mothers at five years of child age whether as symptoms or formal diagnoses. Mothers were asked to answer “Has your child ever had this condition?” for the following (n = 16) using “Yes = 1” or “No = 0” responses: ear infections, rashes or skin problems, meningitis, seizures, high fevers (103 °F or 39 °C or higher), pneumonia, respiratory/breathing problems (other than colds/flu), slow weight gain, trouble with hearing, trouble with seeing, bowel problems, head injury, food allergies/allergy, other allergies/allergy, anemia (low blood count), and kidney or urinary problem(s). A total score of physical health conditions was produced by summing the values of the questionnaire items, resulting in a possible range of zero to 16. All data for respective physical health conditions are provided in Table 2. For each physical health condition, a value of zero was provided if children did not experience any questionnaire item for each respective condition and a value of one was provided if children experienced at least one health experience for the respective physical health condition. Children's physical health conditions were grouped into domain-specific categories (e.g., immune, respiratory) based on conceptual alignment with underlying physiological systems, consistent with approaches used in pediatric health research.

**Table 2:** Categorization of physical health conditions.

Physical Health Condition	Questionnaire Items
*Non-Psychiatric Neurological*	Meningitis, seizures, and head injury
*Respiratory*	Pneumonia and respiratory/breathing problems (other than colds/flu)
*Sensory*	Trouble with hearing and trouble with seeing
*Digestive*	Bowel problems and slow weight gain
*Immune*	Food allergies/allergy, other allergies/allergy, ear infections, high fevers (103 °F or 39 °C or higher), and rashes or skin problems
*Renal*	Kidney problems
*Hematologic*	Anemia (low blood count)
*Total*	Includes all questionnaire items (n = 16)

**Covariates:** Covariates included in this analysis were gestational age, birthweight, sociodemographic characteristics (e.g., maternal highest level of education attained, maternal age at enrolment, total household annual income, self-identified ethnicity), child sex, maternal depressive symptoms, maternal social support, and maternal positive parenting. Gestational age and birthweight were provided by mothers and validated using birth records; if a discrepancy was present, birth record data were used. Sociodemographic characteristics were self-reported by mothers at delivery and child sex was assigned-at-birth.

Maternal depressive symptoms were measured using the Edinburgh Postnatal Depression Scale (EPDS) [32]. The EPDS is a valid and reliable 10-item, 4-point Likert scale, and self-reported measure of depressive symptoms that is commonly used to screen for depression in patients [32]. Scores can range from 0 (no depressive symptoms) to 30 (high levels of depressive symptoms), where greater scores indicate more depressive symptoms [32]. Maternal depressive symptoms were assessed in each trimester of pregnancy and at 3, 6, and 12 months postpartum, and averaged across all time points for analysis. Mothers’ perceived social support was assessed in the first two trimesters of pregnancy and at 3, 12, 24, and 36 months postpartum using the four-item Social Support Questionnaire (SSQ) from the Canadian Community Health Survey [33], and averaged across time points for analysis. This questionnaire evaluates four aspects of social support: informational, emotional, affirmational, and instrumental.^33(p194)^ Responses are recorded on a five-point Likert scale (0 = none of the time, 4 = all of the time), yielding scores between 0 and 16; higher scores indicate greater perceived social support.^33(p194)^ Questions pertaining to positive interactions between parents and children were derived from the Positive Interaction subscale of the Parenting Scale of the National Longitudinal Survey of Children and Youth (NLSCY) at 5 years postpartum [34]. This 5-item, four-point Likert scale, subscale produced a total score ranging from 0 to 20, with higher scores indicating more positive interactions [34].

All covariates were selected a priori based on theoretical and empirical relevance to child physical health outcomes rather than statistical significance. Variance inflation factors were examined in the fully adjusted model and did not indicate problematic multicollinearity among covariates (all variance inflation factors were below 2). Since identical covariates were used across models, multicollinearity was assumed similar for all outcomes.

### 2.4 Statistical analysis

Descriptive statistics, including means, standard deviations, ranges, and proportions, were used to characterize the exposure, outcome, and covariate variables. For the association between mothers' ACEs and their preschool children's total physical health conditions, a negative binomial regression was employed since the data represented count data. For each of the binary physical health concern subtypes, unadjusted and adjusted logistic regression models were employed. In adjusted analyses, cases with missing data for the covariates were automatically deleted through listwise deletion. To best capture the precision and magnitude of reported estimates, confidence intervals (CI) were reported. All analyses were initially conducted with 95% confidence (significance level of 0·05) using R version 4.2.2; however, to correct for multiple comparisons in each model, the significance level was divided by 8 (the number of physical health concern groups), resulting in a CI of 99·38% (or a significance level of 0·00625).

## 3 Results

Demographic characteristics for the sample (n = 540) used in unadjusted analysis are provided (Table 3). This sample constituted roughly equal proportions of female and male children (Table 3). Mothers were mainly of white ethnicity, reporting high educational attainment and total annual household income (Table 3). Most mothers (55·19%) experienced no ACEs (Table 3). The most prevalent physical health concern in children was immune system related (74·81%), while the least prevalent was renal conditions (2·41%; Table 3). The median number of total physical health conditions experienced by children was two (Table 3). For adjusted analyses, complete cases (i.e., those with available data for all covariates) were available for 520 individuals.

**Table 3:** Descriptive statistics of sample (n = 540).

Variables	Frequency
**Sociodemographic Variables**	

*Child Sex-Assigned-at-Birth, n (%)*	
Female	252 (48·46)
Male	268 (51·54)
*Self-Identified Ethnicity, n (%)*	
White	466 (89·62)
Black	6 (1·15)
Asian (i·e·, Chinese, Korean, Southeast Asian, South Asian, Filipino)	30 (5·77)
Indigenous	1 (0·00)
Latin American	11 (2·12)
Middle Eastern (i·e·, Arab)	0 (0·00)
Other (Self-Selected)	6 (1·15)
Missing	2 (0·37)
*Education, n (%)*	
Completed Postgraduate Degree	133 (25·58)
Completed University	263 (50·58)
Completed Technical/Trade School	92 (17·69)
Completed Highschool Diploma or Less	32 (6·15)
Missing	3 (0·56)
*Household Total Annual Income in CAD $, n (%)*	
Less than 20,000	3 (0·58)
20,000 to 39,999	15 (2·88)
40,000 to 69,999	52 (9.63)
70,000 to 99,999	135 (25·96)
100,000 or more	315 (60·58)
Missing	9 (1·67)
*Gestational Age at Birth (weeks)*	
Mean (SD; Range)	39·30 (1·65; 26·43-42·00)
Missing (n, %)	2 (0·37)
*Childbirth Weight (grams)*	
Mean (SD)	3387.25 (510·75)
Min, Max	840·00, 4820·00
Missing (n, %)	3 (0·56)
*Maternal Age at Enrolment (years)*	
Mean (SD)	32·36 (3·82)
Min, Max	22·00, 43·00
*Maternal Social Support*	
Mean (SD)	14·28 (1·96)
Min, Max	5·17, 16·00
*Maternal Depressive Symptoms*	
Mean (SD)	4·69 (3·04)
Min, Max	0·00, 16·33
Missing (n, %)	1 (0·19)
*Positive Parenting*	
Mean (SD)	14·77 (2·52)
Min, Max	8·00, 20·00
Missing (n, %)	6 (1·11)

**Exposure Variable**	

*Maternal Adverse Childhood Experiences*	
0	298 (55·19)
1	114 (21·11)
2	63 (11·67)
3	30 (5·56)
≥4	35 (6·48)

**Outcome Variables**	

*Non-psychiatric neurological, n (%)*	38 (7·04)
*Respiratory, n (%)*	99 (18·33)
*Sensory, n (%)*	62 (11·48)
*Digestive, n (%)*	113 (20·93)
*Immune system, n (%)*	404 (74·81)
*Renal, n (%)*	13 (2·41)
*Hematologic, n (%)*	15 (2·78)
*Total, median (interquartile range)*	2 (2·00)

### 3.1 Unadjusted analysis

Through unadjusted logistic regression analysis (n = 540) and at 95% CI, maternal ACEs were only associated with children's hematologic conditions (OR = 1·37, 95% CI [1·06, 1·72]; Table 4). For every one unit increase in mothers' ACEs, preschool children were 37% more likely to experience hematologic conditions. After correcting for multiple comparisons, mothers' ACEs were no longer associated with children's hematologic conditions (99·38% CI [0·95, 1·87]). However, the 99·38% CI was quite broad, suggesting a lack of precision around the estimate. Maternal ACEs were not associated with total physical health conditions in children before (OR = 1·03; 95% CI [0·99, 1·08]) or after (99·38% CI [0·97, 1·10]) correcting for multiple comparisons (Table 4).

**Table 4:** Unadjusted models of maternal ACEs predicting children's physical health conditions.

Physical Health Concern	Odds Ratio	95% Confidence Interval	99·38% Confidence Interval
*Non-psychiatric neurological*	0·97	0·74, 1·20	0·66, 1·30
*Respiratory*	1·06	0·92, 1·22	0·86, 1·29
*Sensory*	0·98	0·80, 1·16	0·73, 1·24
*Digestive*	1·04	0·91, 1·18	0·86, 1·24
*Immune system*	1·07	0·94, 1·25	0·89, 1·33
*Renal*	0·91	0·53, 1·230	0·40, 1·45
*Hematologic*	1·37	***1·06, 1·72***	0·95, 1·87
*Total*	1·03	0·99, 1·08	0·97, 1·10

### 3.2 Adjusted analysis

Through adjusted logistic regression analysis (n = 520) at 95% confidence including mothers' ethnicity, highest level of educational attainment, total annual household income, positive parenting, mean depressive symptom score, mean social support score, and age at enrolment, gestational age at birth, and birthweight as predictor variables, children's hematologic conditions remained significantly associated with maternal ACEs (OR = 1·45, 95% CI [1·10, 1·86]; Table 5). The odds of preschool children experiencing hematologic conditions when exposed to maternal ACEs were 1·45; for every one unit increase in mothers' ACEs, preschool children were 45% more likely to experience hematologic conditions. Maternal ACEs were not associated with any other health condition (Table 5). After correcting for multiple comparisons (99·38% confidence), maternal ACEs were no longer significantly associated with hematologic conditions (99·38% CI [0·98, 2·06]). However, the 99·38% CI was quite broad, suggesting a lack of precision around the estimate. Maternal ACEs were not associated with total physical health conditions in children before (OR = 1·03; 95% CI [0·99, 1·08]) and after (99·38% [0·97, 1·10]) correcting for multiple comparisons (Table 5).

**Table 5:** Adjusted models of maternal ACEs predicting children's physical health conditions.

Physical Health Concern	Odds Ratio	95% Confidence Interval	99·38% Confidence Interval
*Non-psychiatric neurological*	1·01	0·77, 1·27	0·68, 1·38
*Respiratory*	1·06	0·91, 1·23	0·85, 1·30
*Sensory*	0·99	0·80, 1·18	0·73, 1·27
*Digestive*	1·03	0·89, 1·18	0·84, 1·25
*Immune system*	1·07	0·92, 1·25	0·87, 1·31
*Renal*	0·81	0·45, 1·22	0·33, 1·39
*Hematologic*	1·45	***1·10, 1·86***	0·98, 2·06
*Total*	1·03	0·99, 1·08	0·97, 1·10

## 4 Discussion

This Canadian-based study examined associations between mothers' ACEs and their preschool children's physical health conditions. Maternal ACEs were associated with children's hematologic functioning (indexed by anemia) in both unadjusted and adjusted analyses, with consistent direction and magnitude. However, these associations were attenuated after correction for multiple comparisons. Although most associations were null, the magnitude of the association with anemia (odds ratio of 1.45) suggests potential clinical relevance, particularly given that estimates reflect per-unit increases in maternal ACE exposure within a relatively low-risk sample. Findings should nevertheless be considered preliminary and interpreted cautiously. At the same time, the largely null pattern of results may be reassuring, suggesting that maternal ACE exposure in relatively low-risk populations may not substantially increase intergenerational risk of physical health conditions in early childhood.

### 4.1 Hematologic conditions indexed by anemia: Preliminary evidence for an association

Mothers' ACEs appeared to be associated with children's hematologic conditions, specifically indexed by anemia. One possible explanation is the role of chronic stress on red blood cell functioning, such that children's direct and/or indirect exposure to ACEs can lead to lower red blood cell count, iron absorption, and iron utilization, all of which underlie anemia [7]. While the sample included in this current study was of higher socioeconomic attainment, and most mothers reported low exposure to ACEs, it is possible that children were indirectly exposed to stress-related environments, which in turn, affected their red blood cell functioning. Mothers' ACEs may have also negatively impacted children's hematologic outcomes through other mechanisms. For example, mothers' exposure to ACEs may have intergenerationally influenced their preschool children's red blood cell functioning through the placental transfer of epigenetic markers during pregnancy which decrease red blood cell efficiency [7]. Since a maternal ACE was associated with a 45% increase in anemia in adjusted analysis, this represents a potentially meaningful finding that may contribute to the emerging literature on intergenerational physical health, particularly in relation to anemia.

### 4.2 Null findings

Mothers' ACEs were not associated with their preschool children's non-psychiatric neurological, respiratory, sensory, digestive, immune system, and renal physical health conditions, as well as total physical health conditions. Overall, findings from adjusted analyses are not fully consistent with prior research, where some significant associations between mothers' ACEs and children's respiratory (i.e., asthma) [15,16,18,20], sensory [19], digestive [17,20], and immune-related (e.g., allergies, dermatitis, infections) [15,18], alongside overall physical health conditions [13,14], have been observed. Although prior research suggests that maternal ACEs may influence offspring health through stress-related biological pathways (e.g., dysregulation of cortisol and inflammatory processes), these hypothesized associations were not supported in the current study. Several explanations may account for these null findings, including the relatively low-risk nature of the sample, potential buffering effects in the postnatal environment, and the possibility that associations between maternal ACEs and child physical health may emerge later in development. Even if null, findings from this study contribute to the limited research on these associations and provide novel findings for certain physical health conditions (i.e., non-psychiatric neurological, renal).

### 4.3 Limitations and strengths

This study was comprised of high sociodemographic homogeneity, particularly white ethnicity, educational attainment of at least an undergraduate degree, and a total household annual income equal to or greater than $100,000 CAD, limiting generalization to other demographic groups. However, findings may be more applicable to populations with similar sociodemographic characteristics and contexts. Selective attrition bias likely magnified this limitation, as families experiencing lower socioeconomic attainment are less likely to remain engaged in longitudinal studies.

Although maternal ACEs precede children's physical health outcomes conceptually, both exposure and outcome were measured retrospectively, which may introduce reporting bias and limit the ability to establish clear temporality. Additionally, for children's physical health outcomes, it is not possible to verify whether mothers reported children's conditions based on their own assessment of symptoms or physician diagnoses, as these measures reflect maternal report rather than clinically verified diagnoses. As such, some reported conditions (e.g., ear infections) may represent common childhood experiences rather than clinically verified diagnoses and introduce misclassification bias. While maternal ACEs were measured at 12 months postpartum using the widely used ACEs Questionnaire [[29], [30], [31]], [[29], [30], [31]] children's physical health conditions occurring between 0 and 5 years of age were reported retrospectively at 5 years. As such, temporality is partially inferred rather than directly observed, and findings should be interpreted as associative. The analysis is strengthened by the inclusion of important covariates known to be implicated in children's physical health outcomes, minimizing potential extraneous effects on estimates.

### 4.4 Conclusions

This study provides novel evidence to the field of intergenerational mother-child health by examining hypothesized associations between maternal ACEs and a range of preschool children's physical health conditions. In both unadjusted and adjusted analyses, maternal ACEs were associated with children's hematologic (indexed by anemia) conditions, while no associations were observed for non-psychiatric neurological, respiratory, sensory, digestive, immune system, and renal physical health conditions, as well as total physical health conditions. Although the association with hematologic conditions did not remain significant after correcting for multiple testing, findings suggest that maternal ACEs may be relevant to anemia risk in children, particularly in populations with greater variability in maternal ACE exposure.

Given that diverse mechanisms underlie each physical health concern examined, future research should investigate potential mediation pathways, including biological markers such as cortisol, inflammatory markers, metabolites, and reactive oxygen species. Future work may also consider how emerging contextual factors, including social media and artificial intelligence, shape maternal stress and health during pregnancy, with downstream implications for children's physical health outcomes. Overall, findings should be interpreted as preliminary and hypothesis-generating, given their sensitivity to multiple testing correction and the observational nature of the data. At the same time, results highlight a potential link between maternal ACEs and children's hematologic conditions (indexed by anemia) and suggest that maternal ACEs may not confer substantial intergenerational risk for other physical health conditions in similar low-risk populations.

## Ethical statement

Ethics was obtained from the University of Calgary Health Research Ethics Board (REB14-1702) and the University of Alberta Health Research Ethics 11 Biomedical Panel (Pro00002954).

## Funding

Authors received funding from Alberta Children’s 10.13039/501100022917Hospital Foundation, 10.13039/501100009192Alberta Innovates 10.13039/100018696Health Solutions Foundation, Canadian Institutes for 10.13039/100005622Health Research, 10.13039/501100015741Kids Brain Health Network, and Allergen National Centre of Excellence.

## Declaration of competing interest

The authors declare the following financial interests/personal relationships which may be considered as potential competing interests: Nicole Letourneau reports financial support was provided by Alberta Children’s Hospital Foundation, Alberta Innovates Health Solutions Foundation, Canadian Institutes for Health Research, Kids Brain Health Network, and Allergen National Centre of Excellence. If there are other authors, they declare that they have no known competing financial interests or personal relationships that could have appeared to influence the work reported in this paper.
